# Dental calculus: A repository of bioinformation indicating diseases and human evolution

**DOI:** 10.3389/fcimb.2022.1035324

**Published:** 2022-12-12

**Authors:** Qinyang Li, Kaihua Luo, Zhifei Su, Fangting Huang, Yajie Wu, Fangjie Zhou, Yuqing Li, Xian Peng, Jiyao Li, Biao Ren

**Affiliations:** ^1^ State Key Laboratory of Oral Diseases, National Clinical Research Center for Oral Diseases, Department of Cariology and Endodontics, West China Hospital of Stomatology, Sichuan University, Chengdu, China; ^2^ State Key Laboratory of Oral Diseases, National Clinical Research Center for Oral Diseases, West China Hospital of Stomatology, Sichuan University, Chengdu, China

**Keywords:** dental calculus, bioinformation, disease, anthropology, microbiome

## Abstract

Dental calculus has long been considered as a vital contributing factor of periodontal diseases. Our review focuses on the role of dental calculus as a repository and discusses the bioinformation recently reported to be concealed in dental calculus from three perspectives: time-varying oral condition, systemic diseases, and anthropology at various times. Molecular information representing an individual’s contemporary oral health status could be detected in dental calculus. Additionally, pathogenic factors of systemic diseases were found in dental calculus, including bacteria, viruses and toxic heavy metals. Thus, dental calculus has been proposed to play a role as biological data storage for detection of molecular markers of latent health concerns. Through the study of environmental debris in dental calculus, an overview of an individual’s historical dietary habits and information about the environment, individual behaviors and social culture changes can be unveiled. This review summarizes a new role of dental calculus as a repository of bioinformation, with potential use in the prediction of oral diseases, systemic diseases, and even anthropology.

## Introduction

Dental calculus is the plaque and sediment that has calcified or is calcifying on the tooth surface or prosthodontic body. It can be divided into supragingival calculus and subgingival calculus according to the location of deposition above or below the boundary of the gingival margin (Akcalı and Lang, 2018). Calculus is formed by mineral salts, and it has been reported that the main crystal form of calculus is calcium phosphate, including octacalcium phosphate, hydroxyapatite, whitlockite and dicalcium phosphate dihydrate (Gron et al., 1967). In addition to inorganic components, there are organic components such as proteins and carbohydrates within it, and the outer layer of calculus is always covered with viable plaque.

Bioinformatics, as related to genetics and genomics, is a scientific subdiscipline that involves using computer technology to collect, store, analyze and disseminate biological data and information, such as DNA and amino acid sequences or annotations about those sequences. By using databases that organize and index such biological information, scientists and clinicians can better understand health and disease. Dental calculus has long been considered as one of the contributing factors of periodontal diseases, while plant phytoliths in dental calculus found by Armitage in 1975 indicated its potential as a biological information database (Armitage, 1975). And with the development of genomics, dental calculus, a relatively stable repository of absorbates potentially as a bioinformation database, has become a research hotspot in both archaeological research and modern etiological research since 2013 (Adler et al., 2013). According to these studies, the components deposited in dental calculus seem to be clues that reflect different states oral cavity or other organs. Given that the formation of dental calculus includes the process of absorbing calcium and phosphate from saliva or crevicular fluid over time, it is not surprising that under a microscope, dental calculus has a lamellar structure with absorbates, including oral bacteria and its virulence factors, human proteins, viruses, toxic heavy metals, environment debris and food remnants, deposited layer by layer. Thus, the deposition of dental calculus may also reveal information on the time dimension.

Our review is the first to conclude both the modern and the ancient dental calculus, aiming to summarize the potential role of dental calculus as a “storage library” in the past few years, hoping to provide a new insight to depict the long process of development of diseases and human evolution.

### Key information about oral diseases and time-varying oral conditions in dental calculus

Many archaeological studies have revealed that calculus performs a long-term repository of ancient microbial and host biomolecules because DNA from the oral microbiome can be deposited in dental calculus during its formation. As an indicator, such molecular information, which could be acquired from advanced biomolecular detection methods such as metagenomics, metaproteomics and metabolomes, may provide molecular information about the oral health status of individuals (Warinner et al., 2015; Weyrich et al., 2015; Velsko et al., 2017; Mackie et al., 2017; Jersie-Christensen et al., 2018; Wright et al., 2021). In addition, pathogenic bacteria related to oral diseases such as caries and periodontitis and their virulence factors were also reported to be well preserved in dental calculus. To deduce whether the oral condition was diseased, identifying a signature of specific oral pathogens in calculus became a widely acceptable choice (Bravo-Lopez et al., 1812; Gupta et al., 2016; Willmann et al., 2018; Neukamm et al., 2020). Christina Warinner et al. identified several putative opportunistic pathogens such as *Streptococcus mutans* associated with dental caries and “Red Complex” involving *Porphyromonas gingivalis, Treponema denticola and Tannerella forsythia* linked with periodontitis in ancient dental calculus which could date back to c. 950-1200 CE (Warinner et al., 2014). Compared with samples in the Human Microbiome Project healthy cohort, “Red Complex” were found at substantially higher frequency in ancient dental calculus. Meanwhile, the group also detected both the virulence gene and protein product of “Red Complex” such as Msp/major sheath protein in *T. denticola* and Rgp/Arg-gingipain in *P. gingivalis* and reconstructed genome of *T.forsythia* based on 16S rRNA gene data from ancient dental calculus.Besides, Christensen’s research on medieval samples revealed an unhealthy oral state by detecting the dysbiotic oral microbiome in calculus, which exhibited a number of peri-pathogenic genera and virulence factors from the red complex (Jersie-Christensen et al., 2018). The same group also paid attention to the comparison among ancient calculus, modern calculus and modern plaque. And they found out that ancient calculus and modern calculus could not be classified from each other but they could be separated from modern plaque by “Red Complex” members *P.gingivalis* and *T.forsythia (* Velsko et al., 2019). Moreover, Willmann et al. successfully identify characteristic pathogens responsible for carious, periapical or periodontal diseases presenting in bacterial communities from individuals by combining macroscopic and radiologic analyses with metagenomic analyses (Willmann et al., 2018).

In addition, the information in the calculus showed temporal variations. Some studies on archaeal paleomicrobiology of dental calculus revealed a secular core-microbiota transition in accordance with human evolution, including shifts in dietary, social, or geographic changes in populations (Huynh et al., 2016; Ottoni et al., 2021). At the same time, another study by Eleonora Casula et al. conducted on the samples in the same region of Sardinian Island found out *T. forsythia* was notably higher in modern calculus compared with the ancient. The team attributed the result to antibiotics usage and the relation with systemic diseases such as cardiovascular diseases in addition to dietary changes (Casula et al., 2022). Based on this confirmed role of preserving long-term transition information in calculus, we infer that dental calculus can also function as a database which has the ability to record an individual’s time-varying oral conditions throughout one’s whole life. While the oral microbiome reflects one’s current oral condition, the dental calculus, a more stable substance bonded to teeth, whose formation process is dependent on the oral microbiome, may represent the historical oral condition. Within earlier calculus deposits, remote information can be provided.

### Key information about systemic diseases in dental calculus

According to recent studies, dental calculus can contain oral bacteria, viruses, proteins and small molecules steadily over long periods of time. In the past few years, an increasing number of studies have demonstrated that these contents within dental calculus could be directly linked to several systemic diseases, implying a novel method to trace the causes of the diseases.

Dental calculus entrapped transient bacteria, which may offer us a snapshot of disease exposure (Madhusoodanan, 2016), and particular species known to be involved in the etiology of chronic diseases were detected from metagenomic sequence data of ancient dental calculus. Warinner’s work identified 40 putative opportunistic pathogens in collected dental calculus dated to c. 950-1200 CE that may pose risks of several systemic diseases for the elderly and immunocompromised (Warinner et al., 2014), such as *Streptococcus pneumoniae*, *Streptococcus pyogenes*, *Haemophilus influenzae* related to upper and lower respiratory tract infection and *Aggregatibacter actinomycetemcomitans*, *Streptococcus mutans, Streptococcus mitis* leading to cardiovascular disease risk. Meanwhile, a metagenomic sequencing study on dental calculus from a man died of lobar pneumonia in 1930s St. Louis recovered the genomes of *Klebsiella pneumoniae*, *Acinetobacter nosocomialis*, and *Acinetobacter junii* which may reflect the lobar pneumonia cause of death (Austin et al., 2022).

In addition to pathogenic bacteria, some viruses serving as important roles in systemic diseases can also be traced in dental calculus. By isolating DNA from dental calculus of people diagnosed with oral squamous cell carcinoma (OSCC), the presence of certain human papillomaviruses (HPVs) capable of promoting malignant progression, a verified risk factor for OSCC, was confirmed (Pranata et al., 2020). Likewise, in archaeological research aimed at demonstrating the mouse-man transmission of mouse mammary tumor virus (MMTV), a human MMTV-like betaretrovirus linked with breast cancer was confirmed to be present in ancient dental calculus (Lessi et al., 2020).

Moreover, some studies found novel chemical components in dental calculus connected with systemic diseases. Toxic heavy metals were one of them. Exposure to heavy metals has become a serious health concern in recent decades due to the ubiquity of heavy metals in our daily environment, which may induce a higher risk of cancer in multiple organs. Heavy metals own long biological half-life and can accumulate in dental calculus during calcification (Yaprak et al., 2017; Zhang et al., 2018; Zhang et al., 2019). Toxic heavy metals, including cadmium, arsenic, lead, manganese and vanadium, were detected in dental calculus and were present at significantly higher levels in smokers than in nonsmokers (Yaprak et al., 2017). Another similar study on male OSCC patients with betel-quid chewing habits came up with a similar conclusion. The research indicated remarkably higher cadmium levels in calcified dental calculus samples from patients with habits of betel-quid chewing and smoking compared to healthy individuals without a habit of betel-quid chewing and with smoking. These studies recommended that a non-invasive diagnostic biological material was feasible for monitoring heavy metal exposure and the bioinformation in dental calculus might establish a connection between cadmium and bad habits contributing to increased risk of oral cancer (Zhang et al., 2019). The findings showed that dental calculus may be a vital depositor of information in OSCC and supplied an alternative way for researchers to explore the complex etiology of oral cancer. Moreover, new proteins related to systemic diseases were also found in dental calculus. Lewy bodies and Lewy neurites are the characteristic proteinaceous inclusions in Parkinson’s disease and can also be found in various tissues of the gastrointestinal tract. The protein alpha-synuclein (αSyn), a major constituent of Lewy bodies, was detectable in dental calculus. Although there was a low concentration of αSyn in calculus, it may serve as a referable biomarker, and further studies and advanced detection technology are needed (Schmid et al., 2018).

**Figure 1:**
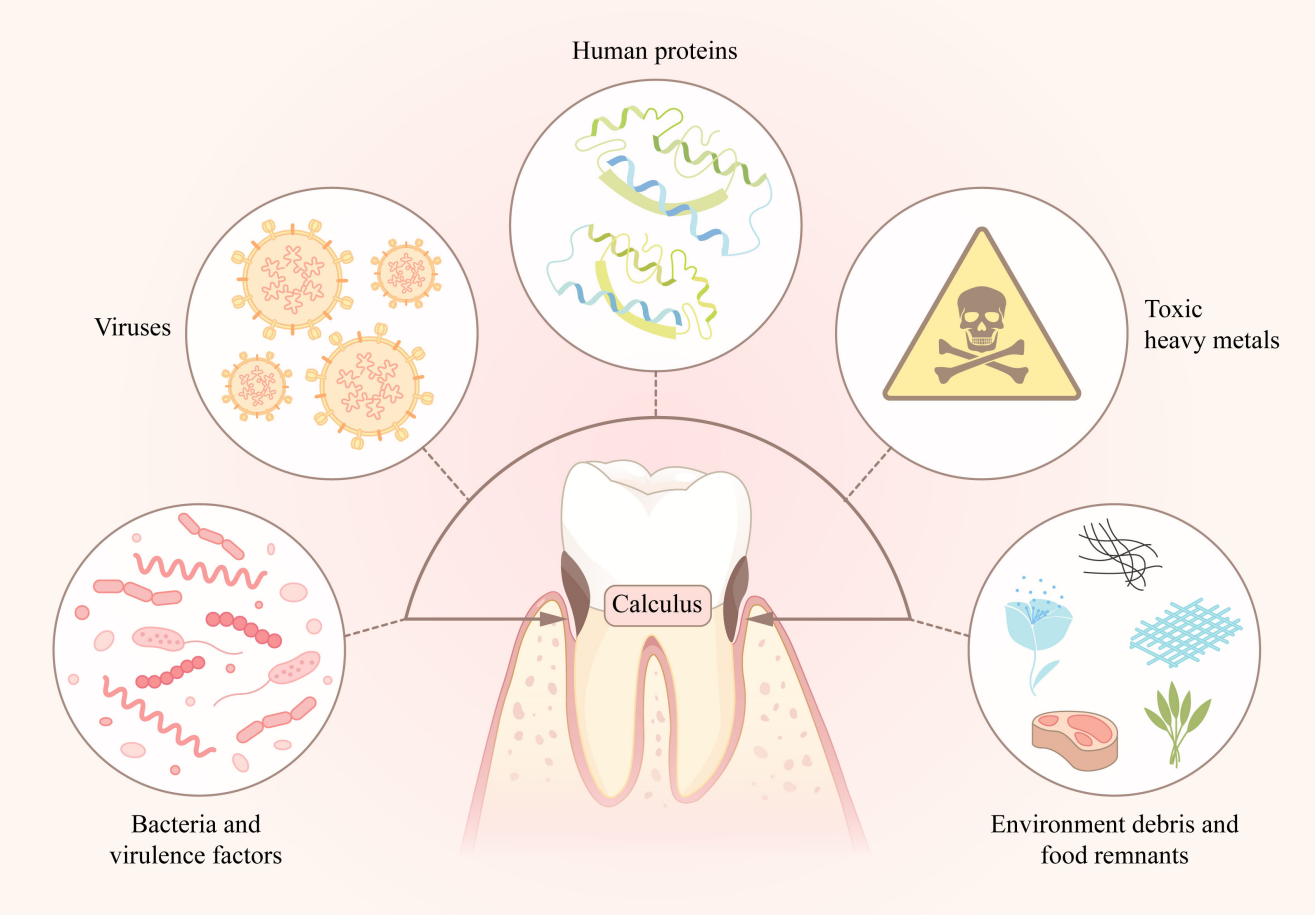
The absorbates in dental calculus, including oral bacteria and its virulence factors, human proteins, viruses, toxic heavy metals, environment debris and food remnants, compose the “storage library”.

Previous studies have supported the idea that there is bioinformation related to some systemic diseases that may be detected in calculus, which inspires us to use another way to find hints about chronic diseases, such as cardiovascular diseases, diabetes, and cancers. Thus, dental calculus may be a potential biological data storage reservoir for detection of molecular markers of latent pathogens, including bacteria, viruses and some protein factors, or other pathogenic factors, such as toxic heavy metals, in patients with systemic diseases (**Figure 1**). To achieve such a function for dental calculus, more studies should be pushed forward. Finding significant biomolecules and detecting them accurately is important for application as detection markers.

### Key information about diet, environment, individual behavior and social culture changes in dental calculus

Dental calculus is in fact a “depositional environment “(deposition of external information) as materials can enter the mouth from a range of sources (Radini et al., 2017). In terms of this aspect, we suppose that calculus contains clues about dietary and environmental information (Hendy et al., 1883; Hardy et al., 2017; Hardy et al., 2018; Sperduti et al., 2018; Demetrowitsch et al., 2020). And through digging out the in-depth knowledge like ancient hominins’ diet, behavior and culture, a new cognition of the long process of human evolution and historical development will be discovered.

Microparticle analysis and stable isotope analysis used to be the most common approaches to study environmental debris in dental calculus. Also, with the development of bioinformatics and advanced sequencing technology in recent decades, more archaeological studies on calculus elucidating hominin dietary habits, behavior and culture have been implemented.

By shotgun sequencing of ancient DNA from Neanderthal dental calculus, Weyrich et al. described the differences in diet matching with the characterization of regional differences in Neanderthal ecology. The Spy Neanderthal diet was primarily meat-based, including woolly rhinoceros and wild sheep (mouflon), which is consistent with the characteristics of a steppe environment (Bocherens et al., 2005). In contrast, the El Sidrón Neanderthal diet contained no meat, while mushrooms, pine nuts, and moss made up the dietary components reflecting forest gathering. Additionally, such differences in diet could give rise to a shift in the hominin’s oral microbiota, might attributed to the meat consumption. Self-medication was also detected in Neanderthals with dental abscesses and chronic gastrointestinal pathogens. And the differences in dietary habits between Spy and the El Sidron neanderthals as well as self-medication suggested that hominin diet and behavior were guided by local environment availability (Fiorenza et al., 2015; Weyrich et al., 2017; Power et al., 2018; Charlier et al., 2019).

Analogously, a recent study on hominins’ dental calculus in the Eastern Alpine region of Italy compares the late paleolithic and mesolithic diet. It provides a more balanced picture of three foragers’ diet, underlining a possible contribution of plant species as food at that time. In particular, starch granules belonging to grass grains, which is of dietary importance, were recovered in the analyzed dental calculus, hence providing the direct evidence that local foragers consumed vegetal resources during their life. Thus, these prehistoric hunter gatherers, as well, were well adapted to the environment in which they lived through exploiting many natural resources (Oxilia et al., 2021). Starch dietary shift takes an important role in human dietary evolution, which is still a major component of the human diet to this day. Moreover, a deep research on dental calculus reveals the link between starch dietary and oral biofilm by reconstructing oral metagenomes and comparing functional adaptations in nutrient metabolism (Fellows Yates et al., 2021). It indicates that drive separation of Homo from nonhuman primates is consistently related to that processing of carbohydrate, largely derived from *Streptococcus*, are much more abundant in Homo. The underlying mechanism can be attributed to the notable ability of *Mitis*, *Sanguinis*, and *Salivarius* groups expressing amylase-binding proteins to capture salivary alpha-amylase, which they use for their own nutrient acquisition and dental adhesion. Alpha-amylase is the most abundant enzyme in modern human saliva, and modern human express it at a higher level than any other hominid. The increase in alpha-amyla has been argued to be associated with dietary shifts during human evolution, specifically an increased reliance on starch-rich foods (Fellows Yates et al., 2021). Lipid, a versatile class of molecule with a broad range of physiological properties and actions, are some of the best-preserved metabolites in historic calculus. A non-targeted assessment of metabolites presented in dental calculus from both modern and historic samples demonstrates the significant potential of calculus as a material for metabolomics and lipidomic studies (Velsko et al., 2017; Velsko et al., 2019).

More abundantly, a variety of debris was detected in the dental calculus sample, including animal micro remains and molecules, hairs, starch granules and other plant micro debris such as fibers and phytochemicals (D’Agostino et al., 2019; D’Agostino et al., 2020). Such an abundant diet indicated that the studied population based its own subsistence on agriculture, husbandry, beekeeping and hunting activities, which also represented proof of the comprehension of food habits, phytotherapeutic practices, and cultural traditions of early colonists (D’Agostino et al., 2019; D’Agostino et al., 2020).

By analyzing historical dental calculus samples, some historical events in the distant past may be reconstructed. To reconstruct the notorious Great Famine of 1845 to 1852, a study used microparticle and proteomic analysis of human dental calculus samples from victims of the famine to elucidate the variability of diet in mid-19th-century Ireland. This study reveals the monotonous potato diet of the poor compared to egg protein of the better-off social classes (Geber et al., 2019). *Via* scanning electron microscopy with energy-dispersive x-ray spectroscopy and micro-Raman spectra, Radini et al. reported the discovery of lapis lazuli pigment preserved in the dental calculus of a religious woman in Germany radiocarbon dated to the 11th or early 12th century, suggesting medieval women’s early involvement in manuscript production (Radini et al., 2019).

Bleasdale’s research utilizing plant microparticles from dental calculus as well as isotope analysis of human and animal remains and charred food remains in Central Africa, spanning the early Iron age to recent history, visually presented new dietary evidence that revealed the long-period variation in the adoption of cereals and the longevity of mosaic subsistence strategies in the region (Bleasdale et al., 2020). And Millard outlined the life-stories of Scottish soldiers experiencing the Battle of Dunbar 1650 from joining the army to their imprisonment by varieties of detection on calculus and bones remains (Millard et al., 2020). We hope that multiple evidence extracted from dental calculus provides unprecedented historical and biographical details for archaeologically recovered individuals and a new insight of process of anthropological evolution.

Dental calculus could record the agelong information and allow us to outline the subsistence pattern of ancient hominins and reconstruct the significant historical event just as happened yesterday with the help of omics and imaging techniques. In view of this opinion, our further study focused on the historical dietary information recorded in the calculus of patients with type II diabetes. In the management and prevention of type II diabetes, dietary factors are of paramount importance (Forouhi et al., 2018). Interestingly, dental calculus may be able to play such a role as a recorder, which records not only food debris or DNA in detail but also metabolic patterns in relation to different diets. As a result, the past dietary patterns of patients with diabetes could be deduced by analyzing one’s calculus, which provides clinicians with a holistic view of the etiological development of disease and allows them to formulate a personalized nutrition approach and guidance for diabetes management.

## Discussion and conclusion

Dental calculus is the calcified plaque or sediment on the tooth surface or prosthodontic body and has long been regarded as the most important local contributing factor of periodontal diseases. Therefore, in clinical treatment, removing this visible risk factor by ultrasonic supragingival scaling and root planning is a key part of initial periodontal therapy (Graziani et al., 2017).

**Figure 2:**
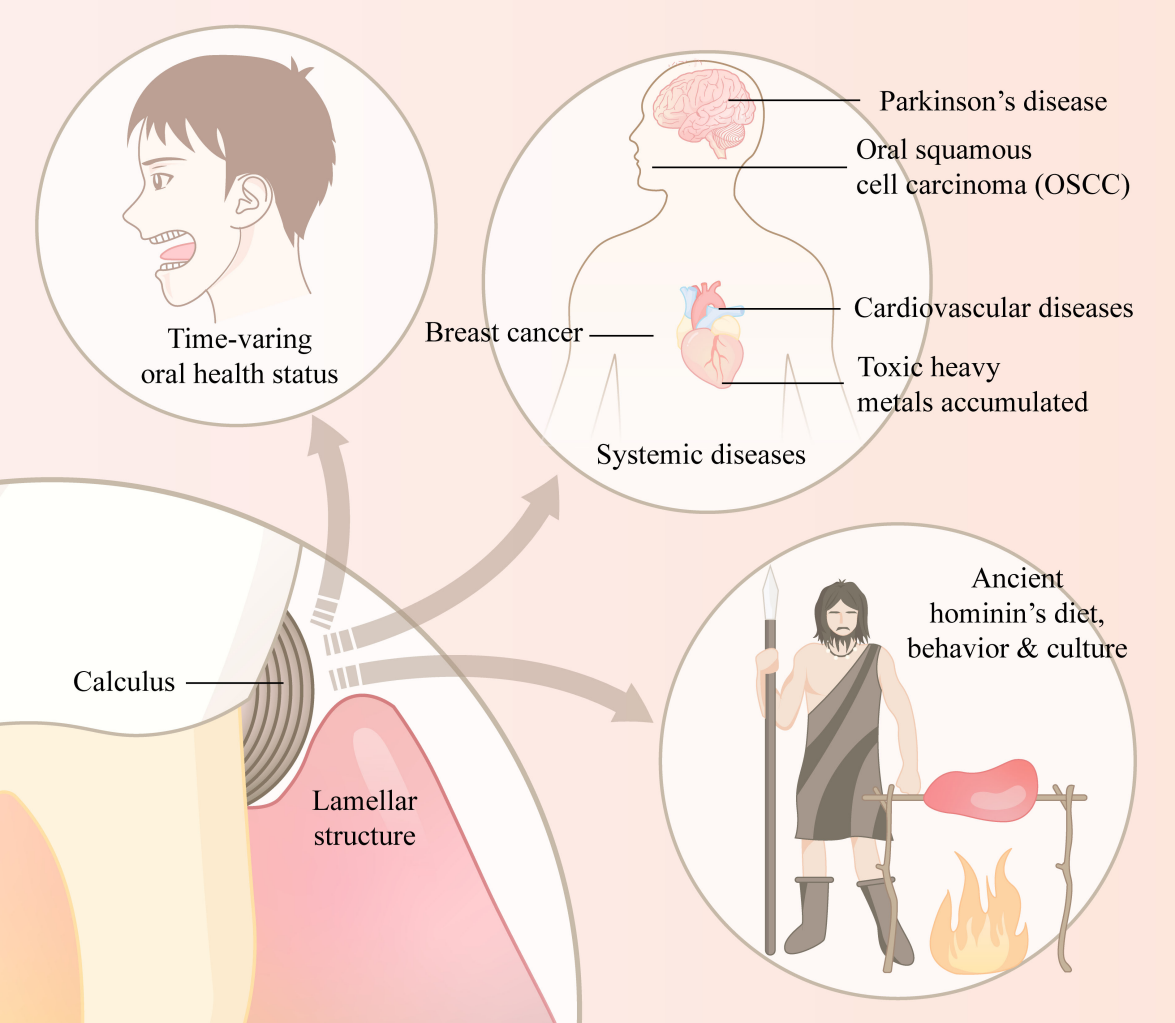
The summary of biological information hidden in absorbates of dental calculus.

In the past few years, dental calculus has become a research hotspot in both archaeological and modern etiological research. By analyzing calculus, a new material in archaeology, archaeologists have provided persuasive inferences about the eating habits, lifestyles and migration changes of people at different times. These findings suggest that calculus can act as a relatively stable repository of bioinformation because the dental calculus used in archaeology usually has a history of hundreds or even thousands of years. Thus, in modern etiological research, dental calculus appears to be reliable for detection. On the one hand, dental calculus can be a code for a state of health or illness, especially for an individual’s oral condition. Using contemporary advanced inspection and analysis technology such as metagenomics, metaproteomics and metabolomics, a comprehensive microbial composition may be achieved and therefore imply that the oral state is healthy. In addition to being a hint for the condition of the oral cavity, dental calculus is also connected with some diseases occurring away from the mouth by means of detecting unique components within it, including viruses, proteins and chemical material. The function as a repository of biological information is illustrated in **Figure 2**. Compared with plaque and saliva, calculus is more difficult to alter by foreign substances or the environment due to its stable crystal properties while they have the ability to store different kinds of information (**Table 1**) Thus, dental calculus seems to be a promising substance to speculate disease information and explore the etiology of distinctive diseases. On the other hand, based on the confirmed role of reserving long-term transition information of human evolution, including shifts in dietary, social, or geographic changes in populations discovered in calculus by archaeologists, we hypothesize that dental calculus also has the ability to record an individual’s time-varying physiological or pathological conditions throughout life in some fields. Like the rings of a tree, calculus may contain information corresponding to time. Thus, when we compare archaeological and modern calculus to understand the human oral microbiome, we’re intended to find similarities and differences, as well as the transition of core-microbiome, hoping to explore the cause of the stabilization and alteration with the help of other bioinformation analyzed from dental calculus such as unique macromolecular substances. The development and wide application of genomics, proteomics and metabolomics help us reveal the hidden bioinformation in the dental calculus. However, for ancient calculus, the degradation of certain substances, uncertain biomolecular preservation and pollution from external environment of long time may lead to the inaccuracy of the result. Therefore, conducting an assessment of preservation and ensuring enough samples are of vital importance in a study. And for modern calculus to diagnose diseases, we need more studies to figure out its accuracy compared with other methods and it’s necessary for us to focus more on the changes in abundance of pathogenic bacteria. In addition, many studies ignore bacterial activity for that the sequencing data is the appearance of DNA so that it’s not reliable to give a comprehensive conclusion only depending on the sequencing data. Dental calculus is the calcified formation of oral biofilm, thus whether the virulence of pathogenic bacteria stay the same or not require more further studies in the future and some *in vitro* experiments and cultures are necessary.

**Table 1:** Comparison of biological components in dental calculus, saliva, and plaque.

	Owned by all	Unique
Dental Calculus	Bacteria, viruses and proteins (Warinner et al., 2014; Warinner et al., 2015., Akcali et al., 2018) toxic heavy metals (Yaprak et al., 2017; Zhang et al., 2018; Öner et al., 2020) immune factors [Cole et al., 1981, Warinner et al., 2014] food debris [Muñoz-González et al., 2018, D’Agostino et al., 2019, D’Agostino et al., 2020]	the ancient DNA of bacteria and hominins, ancient environment debris (including molecules, hairs, starch granules, fibers and phytochemicals) (Warinner et al., 2014; Warinner et al., 2015; D’Agostino et al., 2019; D’Agostino et al., 2020)
Saliva		several biomarkers indicating diseases (like adrenomedullin, nitric oxide, complement C3) [Castagnola et al., 2017], tumor-specific DNA [Zhang et al., 2016]
Plaque		Biofilm structure [Borisy et al., 2021]

## Author contributions

Conceptualization/Methodology, QL, KL, YL, XP, BR, and JL; Writing-Original Draft. Preparation, QL, KL, ZS, FH, YW, FZ; Writing-Review and Editing, QL, KL, ZS, FH, YW, FZ, YL, and XP; Supervision, BR and JL; Project Administration, BR and JL. All authors contributed to the article and approved the submitted version.
